# Application of Biomarkers in Obese Infertile Women: A Genetic Tool for a Personalized Treatment

**DOI:** 10.3390/jcm13082261

**Published:** 2024-04-13

**Authors:** Charalampos Voros, Kyriakos Bananis, Angeliki Papapanagiotou, Abraham Pouliakis, Konstantina Mavriki, Ioannis Gkaniatsos, Maria Anastasia Daskalaki, Ioannis Prokopakis, Charalampos Tsimpoukelis, Aristotelis-Marios Koulakmanidis, Menelaos Darlas, Sofia Anysiadou, Georgios Daskalakis, Ekaterini Domali

**Affiliations:** 1 1st Department of Obstetrics and Gynecology, ‘Alexandra’ General Hospital, National and Kapodistrian University of Athens, 80 Vasilissis Sofias Avenue, 11528 Athens, Greece; ntina_mrk@hotmail.com (K.M.); giannisgan@hotmail.com (I.G.); ioannisprokopakis@gmail.com (I.P.); tsimpoukelischa@gmail.com (C.T.); aristoteliskoulak@gmail.com (A.-M.K.); mdarlas2110@gmail.com (M.D.); sofanysi@yahoo.gr (S.A.); gdaskalakis@yahoo.com (G.D.); kdomali@yahoo.fr (E.D.); 2 Ealing Hospital, London North West University Healthcare NHS Trust, 601 Uxbridge Road, Southall UB1 3HW, UK; kyriakos.bananis@nhs.net; 3 Department of Biological Chemistry, Medical School, National and Kapodistrian University of Athens, 11527 Athens, Greece; agpana@med.uoa.gr; 4 3rd Department of Obstetrics and Gynecology, Attikon Hospital, National and Kapodistrian University of Athens, Rimini 1, 12462 Chaidari, Greece; apou1967@gmail.com; 5 School of Medicine, European University of Cyprus, Nicosia 2404, Cyprus; md181341@students.euc.ac.cy

**Keywords:** infertility, bariatric surgery, CART peptide, leptin, FSHR polymorphism

## Abstract

This study investigates links between CART and leptin gene expression, FSH receptor Asn680Ser polymorphism, and reproductive hormones in morbidly obese patients under 40 years old, facing infertility, and undergoing bariatric surgery. A total of 29 women were included in this study. A hormonal profile along with detection of CART and leptin gene expression was evaluated before and after bariatric surgery. Additionally, the presence or absence of Asn680Ser of the *FSHR* gene was studied. Following bariatric surgery, a mean reduction in BMI (16.03 kg/m^2^) was observed in all women. FSH levels preoperatively varied significantly among genotypes, with medians of 8.1, 9.5, and 10.3 for individuals without polymorphism, heterozygotes, and homozygotes, respectively (*p* = 0.0408). Post surgery, marginal differences in FSH levels were observed (5.8, 7.1, and 8.2, respectively) (*p* = 0.0356). E2 and LH levels exhibited no significant genotype-based differences pre and post surgery. Presurgical E2 levels were 29.6, 29.8, and 29.6, respectively (*p* = 0.91634), while postsurgical levels were 51.2, 47.8, and 47 (*p* = 0.7720). LH levels followed similar patterns. Our findings highlight bariatric surgery’s positive impact on BMI reduction and its potential connection to genetic markers, hormones, and infertility. This suggests personalized treatments and offers a valuable genetic tool for better fertility outcomes in obese individuals.

## 1. Introduction

For many women with severe obesity, bariatric surgery [1,2] is a first step to change their quality of life, as by reducing the excess weight [3], they significantly decrease the incidence of morbidity–mortality [4,5]. Furthermore, by reducing testosterone and dehydroepiandrosterone sulfate (DHEA-S), and increasing luteal hormone (LH) and follicle-stimulating hormone (FSH), bariatric surgery enhances ovarian function. Follicle-stimulating hormone (FSH) interacts with its receptor (FSHR), which is primarily expressed in granulosa cells, to play a crucial role in the development and regulation of the female reproductive system [6]. It is well known that FSH promotes follicular maturation and development, as well as luteinization and ovulation, which are induced by LH. It was previously believed that FSH only affected gonadal tissues. Recently, several studies have shown that FSHRs are expressed in extragonadal tissues, including bone and fat tissue [7].

The single-nucleotide polymorphisms (SNPs) of gonadotropins and their receptors that may have an impact on ovarian response have been found in a number of gene association studies [8,9]. These studies also include SNPs of the *FSHR* gene. The identification of genetic variants related to ovarian response may improve ovarian stimulation and establish a personalized genotype profile. Different studies support a role for the *FSHR* rs6166 (c.2039A>G, p.Asn680Ser) polymorphism as a biomarker of ovarian response to FSH stimulation [10,11]. The Asn/Asn genotype is the normal one and is associated with normal serum levels of FSH. On the other hand, the Ser/Ser genotype, also known as the wild type, was associated with higher levels of FSH, a higher total dose of gonadotropins during ovarian stimulation, and fewer retrieved oocytes [11,12,13]. Consequently, it is proposed that Ser/Ser is associated with a reduced sensitivity of the FSHR to exogenous FSH.

Leptin modulates the female reproductive tract through endocrine and neuroendocrine mechanisms but may also locally regulate ovarian activity, particularly controlling steroidogenesis [14], folliculogenesis [15], and luteal function [16].

It has been shown that the adverse actions of elevated leptin levels associated with obesity are also mediated through cocaine- and amphetamine-regulated transcript (CART) in the ovary. CART possesses well-studied biological activity in the hypothalamus [17]. Numerous actions of CART peptide, including anorexigenic [18,19], neuroendocrine, and antipsychostimulant effects, have been described in the brain across species [20]. Ovarian CART expression was first reported in the bovine ovary [20], and intraovarian CART expression was shown to be associated with the poor health status of follicles. Moreover, studies of the bovine ovary demonstrated that CART is a potent negative regulator of FSH and IGF-1 actions in vitro [21] and an inhibitor of follicular estradiol production in vivo [22]. Although there are many studies focused on CART, leptin, and FSHR polymorphisms, there is no study that combines the expression of the aforementioned genes and polymorphisms in obese women.

In our study, we investigated the occurrence of Asn680Ser polymorphisms within the FSHR gene, along with the levels of reproductive hormones in the serum on day 3 and the expression of CART and leptin genes. This study specifically focused on individuals with both morbid obesity and infertility. 

## 2. Materials and Methods

A cohort of 29 people is included in this study, which strictly abides by the Helsinki Declaration’s ethical requirements. Each and every participant has given their informed consent, demonstrating the transparency and voluntariness of their participation. The scientific boards of the Medical School of Athens (approval no. 48859, date: 30 October 2020) and Alexandra General Hospital (approval no. 4345, date: 1 April 2023) both provided their ethical recommendations.

In this prospective cohort study, the focus is on exploring variations in CART and leptin expression within a specific subset of women facing obesity-related infertility, all below the age of 40. The investigation spans a short period before and six months after sleeve gastrectomy. Infertile patients are identified as those unable to achieve a clinical pregnancy after 12 months of regular, unprotected sexual intercourse. This study was realized at the gynecological tertiary care center of ‘Alexandra’ General Hospital, spanning the period from March 2022 to November 2023.

**Figure 1:**
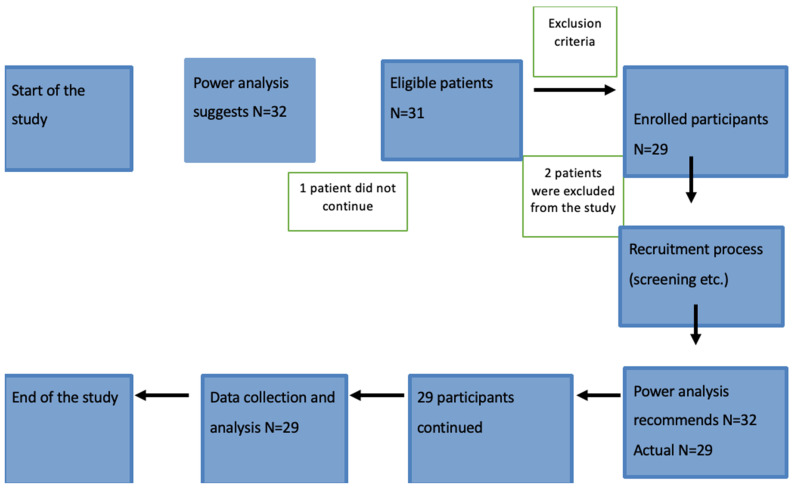
Flow chart of selection process for study individuals.

Our research investigates a certain population of people who are obese and having trouble becoming pregnant. Participants who considered sleeve gastrectomy as a treatment for their obesity and had a body mass index (BMI) of 40 kg/m^2^ or greater were carefully chosen. Because of this, we could be certain that the people we studied had relevant traits that matched our goals. In order to preserve parity among our cohort, we excluded women who were older than 40, guaranteeing that our findings would only apply to individuals who were still able to procreate. To make the sample group more homogeneous, we also excluded individuals who had serious medical disorders that could have an impact on gene expression or fertility. We also excluded women taking drugs known to affect fertility, such as some hormone treatments, in order to avoid tampering with our data. The flowing chart appears in Figure 1. Following sleeve gastrectomy, patients typically stayed in the hospital for 1–2 days. Subsequently, they embarked on a gradual dietary transition from liquids to solids over the course of several weeks. 

Blood samples were taken from patients for our study both prior to and six months following sleeve gastrectomy. The expression of CART and leptin, as well as other pertinent reproductive hormones like follicle-stimulating hormone (FSH), luteinizing hormone (LH), estradiol (E2), sex hormone-binding globulin (SHBG), anti-Müllerian hormone (AMH), and free testosterone, was measured in these samples. Additionally, the Asn680Ser polymorphism was found. A nighttime fast was followed by an early-morning blood sample collection to ensure uniformity and reduce fluctuations in hormone levels.

Women who met particular criteria were included in our study in order to create a focused and distinct group. Participants had to meet the eligibility requirements, which included being classed as morbidly obese and experiencing infertility, with a body mass index (BMI) of 40 kg/m^2^ or more. Furthermore, in order to treat their morbid obesity, participants had to express their intention to undergo a sleeve gastrectomy. This careful process ensured that women with relevant traits that matched our study goals were included.

## 3. Detection of Leptin and CART Gene Expression

All female participants had their blood drawn both prior to and six months following surgery, and the samples were promptly frozen at −80 °C until RNA extraction was completed. The Monarch Total RNA miniprep kit, supplied by New England Biolabs (Ipswich, MA, USA), was used to extract total RNA from blood samples. Next, 1 μg of the extracted RNA was used to create complementary DNA (cDNA) using the LunaScript RT SuperMix, also from New England Biolabs. We used 5 μL of cDNA for real-time polymerase chain reaction (RT-PCR) to measure the leptin and CART gene expression. Using the Luna Universal qPCR Master Mix from New England Biolabs at a 1× final concentration, all RT-PCR reactions were carried out using a LightCycler 480 Instrument II made by Roche Life Sciences (Basel, Switzerland).

The PCR reaction parameters were as follows: a first denaturation phase at 94 °C for one minute, 40 cycles of denaturation at 95 °C for fifteen seconds, and annealing/extension at 60 °C for thirty seconds. A melting curve study was then carried out to confirm the data’s specificity. We additionally used traditional agarose gel electrophoresis to confirm the amplified product’s size in order to enhance our confidence in the PCR reaction’s specificity. Additionally, for standardization, the G6PD gene was employed as a housekeeping gene. To guarantee the accuracy and dependability of the data, each experiment was performed twice. Additionally, a negative control was added to account for any possible contamination or background signal. The 2^−ΔΔCT^ method was used to calculate the relative mRNA expression levels of the CART and leptin genes. 

## 4. Detection of FSHR Polymorphisms

Using the QIAamp DNA Blood Kit (QIAGEN, Hilden, Germany) and following the manufacturer’s instructions, genomic DNA was extracted from peripheral blood leukocytes. The FSHR gene was amplified using polymerase chain reaction with certain oligonucleotide primers. A final volume of 25 μL was used for the PCR reaction, which contained 1× PCR buffer, 10 μM of each primer, two units of Taq-DNA polymerase (Luna, New England Biolabs (Ipswich, MA, USA)), and 5 μL of DNA template. Initial denaturation at 94 °C for 5 min was followed by 40 cycles of denaturation at 94 °C for 1 min, annealing at 60 °C for 1 min, extension at 72 °C for 1 min, and a final elongation step at 72 °C for 10 min. PCR products were digested with BsrI. Digestion was performed in 10 μL reactions containing 1× reaction buffer, five units of the restriction enzyme, and 8 μL of purified PCR product, incubated at 37 °C overnight. Restriction endonuclease digestion products were visualized in 2.5% agarose gels and photographed. The presence of the Asn680Ser genotype introduces a restriction site for BsrI as it creates the A to G transition. Therefore, three different patterns can be observed: a 520 bp band (for 680 Asn/Asn), a 520 bp and a 413 bp band (for 680 Asn/Ser), and a 413 bp band (for 680 Ser/Ser). 

## 5. Sample Size Determination

An analysis of power was carried out to determine the suitable sample size for our research and to guarantee that the investigation has adequate statistical power to identify significant variations in these results. To determine the effect size of the power analysis, we meticulously reviewed the body of current research and examined pertinent publications. For the two main outcome variables, CART and leptin gene expression, we anticipated a moderate effect size (Cohen’s d = 0.5) based on this literature evaluation and expert consultation. It was thought that this effect size was a conservative estimate that would enable the identification of significant alterations in gene expression. A target statistical power threshold of 80% (1 − β = 0.80) was chosen to offer a strong probability of identifying real effects, should they exist. At 0.05, the significance level (α) was established, signifying a 5% possibility of a type I mistake. The mean values of CART and leptin gene expression levels before and six months after bariatric surgery were compared using paired *t*-tests; non-parametric testing for paired data and the Wilcoxon signed-rank test were employed. Assuming a moderate correlation coefficient of 0.5 based on comparable studies, we also took into account the relationship between the pre- and postsurgery measurements. We performed the power study using statistical software (G*Power, version 3.1.9.7) with these settings. Based on that research, we found that in order to achieve the needed statistical power, a total sample size of 29 individuals (or 32, if we assume a 10% loss of follow-up) would be required. To make sure this study could be conducted in a clinical context, practical factors including patient availability and resources were taken into account.

## 6. Statistical Analysis (Methods)

Data were recorded on Microsoft Excel (version 2401, 2016) spreadsheets (Microsoft Corporation, Redmond, Washington, DC, USA) in rows corresponding to each patient. Statistical analysis was performed via the SAS for the Windows 9.4 software platform (SAS Institute Inc., Cary, NC, USA). Descriptive data were expressed as mean value and standard deviation (SD). We calculated the differences in the levels of hormones as their value after surgery minus the value before surgery (therefore, a positive value is linked with increment and a negative value with reduction); in terms of statistics, we applied non-parametric tests for paired data, specifically the Wilcoxon signed-rank test. The number of patients who experienced an increment or reduction in the hormonal values and the relevant percentages was also calculated. To investigate whether the reduction (or increment) in hormones was linked with a reduction or increment in the BMI as well as to the studied gene expressions, we calculated the Spearman correlation coefficient (rs) (a positive value near to 1 indicates a strong positive correlation, while a negative value nearer to −1 a strong negative role). The significance level for the study was set to *p* < 0.05, and all tests were two-sided.

## 7. Results

This study involved 29 women following bariatric surgery (sleeve gastrectomy) because of morbid obesity, who were monitored 6 months after the operation. Their clinical and biochemical characteristics are shown in Table 1.

Of the 29 individuals, 8 (27.6%) did not have the Asn680Ser polymorphism, 13 (44.8%) were heterozygotes, and 8 (27.6%) were homozygotes. We investigated for possible differentiation in the CART and leptin expression before and after surgery, as well as for the other hormones. Table 2 showing the relevant expressions for the three groups is presented below.

It was found that the change in leptin and CART gene expression before and after the operation was not different among the three groups, *p* = 0.4275 and *p* = 0.6387, respectively. A detailed analysis showed that no difference was found between those without the polymorphism and those detected with the variant. 

According to FSH before surgery, the median for Q1–Q3 for individuals without the polymorphism was 8.1 (7.6–10.1), for the heterozygotes 9.5 (8.8–11.2), and for the homozygotes 10.3 (8.1–11.9), with significant differences (*p* = 0.0408) between the three groups. More specifically, we detected that homozygous individuals without the polymorphism and heterozygous individuals have a significant difference (*p* = 0.0362); the same statistically significant difference is detected (*p* = 0.0292) for homozygotes without the polymorphism and homozygotes with the polymorphism. Postsurgically, the median values were 5.8 (5–6.7), 7.1 (5.6–7.8), and 8.2 (4.7–9.1), respectively, with a marginal difference (*p* = 0.0356). Furthermore, our analysis showed that, between homozygotes without the polymorphism and heterozygotes, there was a statistically significant difference (*p* = 0.0458); the same significance was found for homozygotes without the polymorphism and homozygotes with the polymorphism (*p* = 0.0484). Concerning the FSH difference (i.e., postsurgical minus presurgical), the median was 3.2 (2.7–3.8) and 2.7 (2–3.4), respectively, without a confirmed significant difference (*p* = 0.403866).

When focusing on E2, and comparing the patients without the polymorphism, heterozygotes, and homozygotes, the presurgical median for Q1–Q3 was 29.6 (29.5–32.7), 29.8 (27.1–32), and 29.6 (27.7–31.4), respectively, without a significant difference (*p* = 0.91634). Postsurgically, the median was 51.2 (44.4–54.6), 47.8 (44.8–50.7), and 47 (45.1–51.9), again without detection of a significant difference (*p* = 0.7720). Finally, for the difference in E2 (postsurgical–presurgical), the median was 17.7 (15.2–22.3) and 18.2 (13.7–22.5) for the heterozygotes and homozygotes, respectively, without any difference (*p* = 0.8280).

Similarly, for LH, when comparing women without the polymorphism, heterozygotes, and homozygotes, presurgically, the median for Q1–Q3 was 6.4 (6.2–6.7), 6.8 (6.3–7), and 6.3 (5.9–6.4), respectively, without any significant difference (*p* = 0.3440). Postsurgically, the median values for Q1–Q3 were 9.4 (8.9–9.9), 8.9 (8.6–9.5), and 8.4 (7.7–9); we detected a statistically significant difference (*p* = 0.0325) between homozygotes without the polymorphism and homozygotes with the polymorphism. Finally, for the LH difference (postsurgical–presurgical), the mean was 2.9 (2.1–3.7), 2.7 (2–3.3), and 2.5 (1.2–2.9) for the three groups of individuals, without any difference (*p* = 0.4172).

## 8. Discussion 

Treatment options for obesity encompass a range of strategies, including both non-surgical and surgical approaches. Among these, bariatric surgery emerges as a highly effective choice with promising outcomes, such as diabetes prevention, reduced cardiovascular risk, and obesity-induced infertility [23,24,25]. Our study focused on individuals undergoing sleeve gastrectomy, a procedure involving the removal of a significant portion of the stomach, which alters hormonal balance [26]. This surgical option was selected for its simplicity compared to other bariatric procedures like gastric bypass, and its avoidance of foreign objects within the body [27]. Additionally, sleeve gastrectomy has been associated with decreased production of the hunger hormone ghrelin, potentially curbing appetite in patients [26].

Even though assisted reproductive technologies (ART) offer hope to couples facing infertility, the interplay between obesity and the results of these technologies is of major importance. In the present study, we attempted to identify the possible interactions between BMI change after a bariatric operation, the hormonal profile, and the genetic profile of infertile women. Our research protocol revealed notable disparities in FSH levels among individuals with different genotypes of Asn680Ser polymorphisms. Our research detected significant differences in serum levels of FSH before sleeve gastrectomy among the three genetic groups, especially when homozygotes for the wild-type allele were compared with homozygotes for the polymorphism. However, postsurgical FSH levels showed only marginal differences between the groups. These findings suggest a potential association between genetic polymorphisms and hormonal regulation, particularly in FSH levels, which could influence surgical outcomes and postoperative hormonal balance. Furthermore, in our study, we found that there was a statistically significant difference in serum levels of LH after the intervention between the homozygotes for the allele and the homozygotes without the polymorphism. On the other hand, analyses of E2 levels did not divulge statistically significant differences among the genetic groups either preoperatively or postoperatively. When we compared the three groups, both pre- and postsurgical levels of E2 remained relatively consistent across the different genetic polymorphisms. The lack of significant differences in E2 levels suggests that this hormone may not be as influenced by the genetic variations studied compared to leptin and FSH. Cui et al. showed that preadipocytes extracted from chicken adipose tissue formed adipocyte morphology faster when exposed to FSH, and the FSHR expression was also upregulated [28]. Anagnostou et al. showed in their study that the obese patients (BMI ≥ 30 kg/m^2^) were in need of increased total FSH dose (*p* < 0.001) and more days of stimulation (*p* = 0.002), but their basal FSH levels were lower (*p* = 0.032). Concerning the polymorphism of the FSHR gene, no differences were observed in the genotype distribution between the obese non-PCOS women and the non-obese women [29]. Sudo and collaborators identified a statistically significant distinction in serum FSH levels among various genotypes in their study [30]. Subsequently, in a separate study, de Castro et al. mentioned a higher frequency of IVF cycle cancellations in women with homozygous Ser (Ser/Ser) genotypes. This genotype was more prevalent in poor responders and correlated with a diminished response rate to administered recombinant FSH [31]. Chou and Mantzoros stated that even though leptin concentration has no major role in the hypothalamic–pituitary–gonadal axis and consequently in the regulation of ovarian function, as it is directly correlated with BMI, it may indirectly cause infertility [32]. Wertel et al. showed that the levels of leptin were not associated with the pathophysiology of infertility [33]. Kitawaki et al. revealed that estrogen synthesis is stimulated by leptin by increasing the mRNA and protein expression of P450 aromatase in human granulosa cells [34]. Additionally, several studies found stimulatory effects of leptin on LH and FSH levels [14]. Zuhair et al. concluded that leptin levels were significantly increased with higher BMI in infertile women, while no such correlation was observed in the control group [35]. Wu et al., in their study, investigated the presence of a genetic profile related to obese infertile PCOS patients undergoing IVF-ET. According to their results, weight loss may regulate the hormonal and gene expression profiles of granulosa cells, and consequently improve the ovarian responsiveness, embryo quality, implantation, and clinical pregnancy rates and live births [36]. Kaur et al. proposed that allelic frequencies of Asn680Ser were not significantly different between PCOS women and the control group, but clinical features of PCOS including hyperandrogenism and dyslipidemia were significantly correlated with *FSHR polymorphism* [37]. Chu et al. showed that silencing the receptor of leptin reduced E_2_ concentration and the addition of leptin affected *Cyp19a1*, *Cyp11a1*, and *FSHR* gene expression [38].

Ma et al. reported that, under obese conditions, leptin induces CART expression in the ovary, which leads to adverse effects on ovarian functions and consequently to fertility problems. They showed that although they detected *CART* mRNA in GCs from women with normal BMI, its expression was significantly higher in overweight and obese women. The authors proposed that the increased CART expression in the ovary is detrimental to follicular development and female fertility, suggesting that although CART likely acts as a gatekeeper for follicle selection, too much CART (in the case of obesity) prevents follicles from selection, preovulatory development, and ovulation [39]. Assessing infertility issues in women suffering from infertility through the establishment of a genetic profile provides a useful tool for the prediction of IVF outcome. Additionally, in our study, we combined the study of expression of key genes in obesity regulation and the presence of a highly abundant polymorphism. 

To our knowledge, this is the first study which relates the levels of CART and leptin gene expression with the presence or absence of Asn680Ser polymorphism and the hormonal profile of women undergoing bariatric surgery. Although no statistically significant relationships were proposed between the expression of genes and the polymorphism, a more extended study of all the polymorphisms of the FSHR gene in women with obesity may reveal possible associations and establish a genetic profile.

## 9. Conclusions

Even though assisted reproductive technologies (ART) offer hope to couples facing fertility issues, the interplay between obesity and the results of these technologies is of major importance. In the present study, we attempted to identify the possible interactions between BMI change after a bariatric operation, the hormonal profile, and the genetic profile of infertile women. It was found that the change in leptin and CART gene expression before and after the operation was independent of the presence of the variant. On the contrary, differences in the levels of FSH were revealed regarding the presence or absence of FSHR polymorphism. Moreover, our research has shown that postoperatively, there was a significant difference in the serum levels of LH between the homozygotes for the Asn680Ser and the group without the polymorphism. We expect that with a larger sample of patients, the correlations between the hormone levels and the genotype will also be stronger for the other groups. 

In treating obese infertile women, a multidisciplinary approach involving collaboration among bariatric surgeons, reproductive endocrinologists, geneticists, and other healthcare providers is paramount. The results of our study highlight the intricate interplay between genetic variations (FSR polymorphism Asn680Ser), hormonal profile, and CART and leptin gene expression, offering important data for treatment optimization.

Firstly, clinicians should consider incorporating genetic profiling into the evaluation of obese patients suffering from morbid obesity before suggesting any potential treatment. Genetic variations, such as the Asn680Ser polymorphism of the FSHR gene, may exert a significant impact on hormonal regulation and fertility outcomes, as referred to in recent studies. By identifying whether the polymorphism exists or not, clinicians can give guidance to patients on personalized treatment strategies and anticipate their response to interventions like bariatric surgery or assisted reproductive technologies (ART). According to the literature, individuals harboring the polymorphism have higher levels of serum FSH or they will need larger doses of gonadotropins in stimulating protocols.

The assessment of reproductive hormones levels (such as FSH and LH) before intervention is essential for guiding treatment decisions. Our results suggest that individuals with certain genetic variations may exhibit distinct hormonal patterns, which impact surgical outcomes and postoperative hormonal balance. Moreover, after bariatric surgery, ongoing monitoring is crucial. By tracking postoperative alterations in hormone levels, physicians can detect early signs of imbalances and adjust treatment protocols. This collaborative approach enables proactive management of complications after surgery and optimization of fertility results.

The combination of hormonal assessments and genetic profiling allows for a holistic understanding of individual patient characteristics and informs personalized treatment planning. Educational initiatives aimed at empowering women suffering from morbid obesity with knowledge about their genetic predispositions and hormonal profiles are also very important for fostering informed decision-making and enhancing patient engagement in their care. A future attempt should be made to study extensively the polymorphisms of the FSHR gene and to correlate them with CART and leptin gene expression and with the hormonal profile of obese women suffering from infertility.

## Figures and Tables

**Table 1:** Clinical characteristics of the women included in this study.

Characteristics	Mean ± SD
Age (years)	33.0 ± 4.1 (26, 39)
Weight (kg)	109.89 ± 19.28
Height (m)	1.53 ± 0.21
BMI (kg/m^2^)	41.94 ± 3.98
LH (mIU/mL)	6.35 ± 0.67
E2 (pg/mL)	29.81 ± 2.65
SHBG (nmol/L)	36.24 ± 7.58
Free testosterone (ng/dL)	28.72 ± 9.35
AMH (ng/mL)	2.14 ± 0.24
Duration of infertility (years)	3 (2, 5)
Cpt0 CART (adjusted)	0.3 ± 4.41
Cpt0 leptin (adjusted)	−1.81 ± 1.82

Twenty-nine patients participated in this study; their mean age was 33.0 years (SD: 4.1 years, range: 26–39 years). Their mean weight and height were 109.89 kg (SD:19.28) and 1.53 m (SD: 0.21), respectively. Their BMI was 41.94 kg/m^2^ (SD: 3.98).

**Table 2:** Association of polymorphisms with CART and leptin genes as well as hormones before and after surgery.

	Homozygotes without the Polymorphism (N = 8)	Heterozygotes for the Polymorphism (N = 13)	Homozygotes for the Polymorphism (N = 8)	*p*-Value between the Three Groups	*p*-Value between Homozygotes without the Polymorphism and Heterozygotes with the Polymorphism	*p*-Value between Homozygotes without the Polymorphism and Homozygotes with the Polymorphism	*p*-Value between Heterozygotes and Homozygotes with the Polymorphism
Characteristic	Median (Q1–Q3)	Median (Q1–Q3)	Median (Q1–Q3)	*p*	*p*	*p*	*p*
AFC—Left ovary—before surgery	10 (8.5–11)	8 (7–10)	10 (8–12.5)	0.1775	0.1775	0.8738	0.1252
AFC—Left ovary—post surgery	7 (5.5–8.5)	7 (6–7)	6.5 (6–8)	0.7730	0.5016	0.8724	0.6258
AFC—Left difference	−3 (−4–−2)	−2 (−3–0)	−3.5 (−4.5–−1.5)	0.3088	0.2000	0.7889	0.2145
AFC—Right ovary—before surgery	12 (10–12.5)	9 (8–12)	10.5 (9–11.5)	0.4466	0.3240	0.3105	0.4005
AFC—Right ovary—post surgery	6 (5–9.5)	8 (7–8)	7.5 (7–9)	0.5267	0.5086	0.2875	0.5027
AFC—Right difference	−3.5 (−7–−1.5)	−2 (−4–0)	−2 (−4.5–−0.5)	0.4693	0.2744	0.2897	0.9127
AMH (ng/mL)—before surgery	2.2 (1.9–2.3)	2.2 (2–2.4)	2.2 (1.9–2.4)	0.8592	0.6081	0.8727	0.7155
AMH (ng/mL)—post surgery	3 (2.9–3.1)	3 (2.8–3.1)	2.9 (2.8–3)	0.4507	0.9408	0.1957	0.3323
AMH difference	3.9 (2.7–4.4)	3.2 (2.7–3.8)	2.7 (2–3.4)	0.3460	0.3454	0.1886	0.4039
Age	31 (28–35.5)	36 (35–37)	31.5 (27.5–34.5)	0.0318	0.0680	0.5237	0.0163
BMI (before surgery)	41.8 (39.2–42.7)	41.2 (38.9–44.5)	40.7 (37.9–45.6)	0.9464	0.7173	0.8336	0.8563
BMI (after 6 months)	26.1 (25.3–26.9)	25.7 (24.8–26.2)	26.5 (25.3–27.2)	0.4375	0.6892	0.5271	0.1792
BMI difference	−14.8 (−16.7–−13.8)	−15.3 (−18.8–−14.3)	−13.6 (−19–−11.3)	0.2578	0.4913	0.3446	0.1111
Cpt1-Cpt0 CART	−1.7 (−2.4–−1.3)	−2.1 (−2.6–−1.8)	−1.5 (−12.7–−1.3)	0.6387	0.3848	1.0000	0.5006
Cpt1—Cpt0 leptin	2 (1.6–2.6)	2 (1.4–2.3)	1.7 (0.7–1.9)	0.4275	0.5623	0.2030	0.3832
E2 (pg/mL)—before surgery	29.6 (29.5–32.7)	29.8 (27.1–32)	29.6 (27.7–31.4)	0.6834	0.5141	0.3713	0.9134
E2 (pg/mL)—post surgery	51.2 (44.4–54.6)	47.8 (44.8–50.7)	47 (45.1–51.9)	0.8078	0.6121	0.5286	0.7720
E2 difference	19.7 (15.7–21.8)	17.7 (15.2–22.3)	18.2 (13.7–22.5)	0.9014	0.8563	0.5995	0.8280
FSH (mIU/mL)—before surgery	8.1 (7.6–10.1)	9.5 (8.8–11.2)	10.3 (8.1–11.9)	0.0408	0.0362	0.0292	0.0515
FSH (mIU/mL)—post surgery	5.8 (5–6.7)	7.1(5.6–7.8)	8.2(4.7–9.1)	0.0356	0.0458	0.0484	0.0685
FSH difference	2.6 (2.1–4.0)	2.4 (1.7–3.8)	2.1 (1.6–3.1)	0.3460	0.1454	0.0886	0.2039
Free testo (ng/dL)—before surgery	32.4 (21.2–37.5)	31.5 (27.3–34.3)	25.6 (19.1–29.5)	0.3107	0.6639	0.2936	0.1281
Free testo (ng/dL)—post surgery	9.3 (6.4–11.7)	9.3 (7.4–11.2)	8.9 (8.4–10.7)	0.9431	0.6638	0.9157	0.9133
Free testo difference	−25.4 (−27.7–−10.4)	−21.8 (−26.3–−15.3)	−17.7 (−20.6–−10.1)	0.3548	0.7171	0.2476	0.1922
LH (mIU/mL)—before surgery)	6.4 (6.2–6.7)	6.8 (6.3–7)	6.3 (5.9–6.4)	0.3440	0.3627	0.3963	0.2016
LH (mIU/mL)—post surgery	9.4 (8.9–9.9)	8.9 (8.6–9.5)	8.4 (7.7–9)	0.1011	0.2609	0.0325	0.1466
LH difference	2.9 (2.1–3.7)	2.7 (2–3.3)	2.5 (1.2–2.9)	0.4172	0.3848	0.2936	0.3462
SHBG (nmol/L)—before surgery	40.4 (27.9–46.4)	39.1 (34.5–41.3)	32 (25.9–38)	0.1106	0.8563	0.1415	0.0357
SHBG (nmol/L)—post surgery	69.7 (58.9–81.3)	65.3 (58.7–72.3)	58.2 (49.5–67)	0.2383	0.5380	0.1415	0.1688
SHBG difference	30.9 (23.4–36.6)	28.3 (21.2–34.9)	27.4 (21.9–31.7)	0.7953	0.7172	0.4623	0.7720

## Data Availability

Not applicable.
